# Clinical, biochemical, and genetic features associated with *VARS2*‐related mitochondrial disease

**DOI:** 10.1002/humu.23398

**Published:** 2018-02-07

**Authors:** Francesco Bruni, Ivano Di Meo, Emanuele Bellacchio, Bryn D. Webb, Robert McFarland, Zofia M.A. Chrzanowska‐Lightowlers, Langping He, Ewa Skorupa, Isabella Moroni, Anna Ardissone, Anna Walczak, Henna Tyynismaa, Pirjo Isohanni, Hanna Mandel, Holger Prokisch, Tobias Haack, Penelope E. Bonnen, Bertini Enrico, Ewa Pronicka, Daniele Ghezzi, Robert W. Taylor, Daria Diodato

**Affiliations:** ^1^ Wellcome Centre for Mitochondrial Research Institute of Neuroscience Newcastle University Newcastle upon Tyne United Kingdom; ^2^ Molecular Neurogenetics Unit Foundation IRCCS Neurological Institute C. Besta Milan Italy; ^3^ Genetics and Rare Diseases Research Division ‘Bambino Gesù’ Children Hospital Rome Italy; ^4^ Department of Genetics and Genomic Sciences Icahn School of Medicine at Mount Sinai New York New York; ^5^ Department of Biochemistry Radioimmunology and Experimental Medicine The Children's Memorial Health Institute Warsaw Poland; ^6^ Child Neurology Unit Foundation IRCCS Neurological Institute “C. Besta” Milan Italy; ^7^ Department of Molecular and Translational Medicine DIMET University of Milan‐Bicocca Milan Italy; ^8^ Department of Medical Genetics Centre of Biostructure Medical University of Warsaw Warsaw Poland; ^9^ Research Programs Unit Molecular Neurology University of Helsinki Helsinki Finland; ^10^ Department of Pediatric Neurology Children's Hospital University of Helsinki and Helsinki University Hospital Helsinki Finland; ^11^ Institute of Human Genetics and Metabolic Diseases Galilee Medical Center Nahariya Israel; ^12^ Institute of Human Genetics Technische Universität München Munich Germany; ^13^ Institute of Human Genetics Helmholtz Zentrum München Neuherberg Germany; ^14^ Department of Molecular and Human Genetics Baylor College of Medicine Houston Texas; ^15^ Unit of Neuromuscular and Neurodegenerative Disorders Laboratory of Molecular Medicine ‘Bambino Ges.’ Children's Research Hospital Rome Italy; ^16^ Department of Pediatrics Nutrition and Metabolic Diseases The Children's Memorial Health Institute Warsaw Poland; ^17^ Department of Pathophysiology and Transplantation University of Milan Milan Italy

**Keywords:** cardioencephalomyopathy, mitochondrial disorders, OXPHOS, VARS2

## Abstract

In recent years, an increasing number of mitochondrial disorders have been associated with mutations in mitochondrial aminoacyl‐tRNA synthetases (mt‐aaRSs), which are key enzymes of mitochondrial protein synthesis. Bi‐allelic functional variants in *VARS2*, encoding the mitochondrial valyl tRNA‐synthetase, were first reported in a patient with psychomotor delay and epilepsia partialis continua associated with an oxidative phosphorylation (OXPHOS) Complex I defect, before being described in a patient with a neonatal form of encephalocardiomyopathy. Here we provide a detailed genetic, clinical, and biochemical description of 13 patients, from nine unrelated families, harboring *VARS2* mutations. All patients except one, who manifested with a less severe disease course, presented at birth exhibiting severe encephalomyopathy and cardiomyopathy. Features included hypotonia, psychomotor delay, seizures, feeding difficulty, abnormal cranial MRI, and elevated lactate. The biochemical phenotype comprised a combined Complex I and Complex IV OXPHOS defect in muscle, with patient fibroblasts displaying normal OXPHOS activity. Homology modeling supported the pathogenicity of *VARS2* missense variants. The detailed description of this cohort further delineates our understanding of the clinical presentation associated with pathogenic *VARS2* variants and we recommend that this gene should be considered in early‐onset mitochondrial encephalomyopathies or encephalocardiomyopathies.

## 1 INTRODUCTION

Mitochondria are defined as the powerhouses of the cell, since they produce a usable energy source, adenosine triphosphate (ATP), through oxidative phosphorylation system (OXPHOS). This process is carried out by the mitochondrial respiratory chain (MRC), and the ATP synthase, which are embedded in the inner mitochondrial membrane (MIM). The MRC is composed of four multi‐subunit complexes (CI to CIV) and two mobile electron carriers (ubiquinone and cytochrome *c*) that produce a proton gradient across the MIM, which is then used by Complex V (FoF1 ATP synthase) to produce ATP [Lightowlers, Taylor, & Turnbull, 2015; Smeitink, Zeviani, Turnbull, & Jacobs, 2006]. OXPHOS proteins are uniquely under the dual genetic control of two genomes and as such, mitochondrial disorders can be caused by defects in either the mitochondrial DNA (mtDNA) or nuclear DNA [Spinazzola & Zeviani, 2009]. Mutations in several genes responsible for defects of mitochondrial protein synthesis, affecting either mtDNA or nuclear encoded genes, have been reported to cause a wide range of mitochondrial syndromes [Ghezzi & Zeviani, 2012; Rotig, 2011]. Mitochondrial aminoacyl‐tRNA synthetases (mt‐aaRSs) are key enzymes in mitochondrial protein synthesis since they catalyze the specific attachment of amino acids to their cognate tRNAs. Recently, an increasing number of mitochondrial disorders have been associated with variants in mt‐aaRSs genes [Coughlin et al., 2015; Diodato et al., 2014ba,a; Konovalova & Tyynismaa, 2013; Simon et al., 2015]. However, for some mt‐aaRSs (i.e., VARS2, MIM# 615917), very few individual patients have been described and the associated phenotype is thus poorly defined. A homozygous missense pathogenic variant (c.1100C > T, p.Thr367Ile; NM_001167734.1) in *VARS2*, the gene encoding the mitochondrial valyl tRNA‐synthetase, was described in a patient with a clinical picture characterized by psychomotor delay and epilepsia partialis continua associated with a mitochondrial Complex I defect [Diodato et al., 2014ba,b]. Additionally, compound heterozygous variants in *VARS2* (the previously reported c.1100C > T, p.Thr367Ile plus a c.601C > T, p.Arg201Trp variant) were reported in a patient with a neonatal form of encephalocardiomyopathy [Baertling et al., 2017]. Pathogenic variants in *VARS2* were also described in two patients included in large whole exome sequencing (WES) studies although detailed clinical and/or biochemical characterization was not provided [Pronicka et al., 2016; Taylor et al., 2014]. Here we report a detailed clinical and molecular description of the patients (and affected siblings) reported in the previous WES studies and describe 11 additional patients from unrelated families presenting with a severe infantile mitochondrial disorder associated with *VARS2* pathogenic variants, further defining the clinical and biochemical features associated with *VARS2* defects.

## 2 MATERIALS AND METHODS

### 2.1 Standard protocol approvals and patient consent

Informed consent to participate in the study was obtained from all affected individuals or their parents in case of study participants under the age of consent. The study was approved by the Ethical Committees of the Institutional Review Board at the Icahn School of Medicine at Mount Sinai, New York, NY (P1 and P2), the Technische Universität München (P3), the Neurological Institute Besta, Milan, Italy (P4), the National Research Ethics Committee (UK) (P5), the Bioethical Commission at Children's Memorial Health Institute (P6‐7), the Ethical Committee of Baylor College of Medicine, Houston, US (P8‐10), and the Ethical Review Board of Helsinki University Hospital, Helsinki, Finland (P11‐13) in agreement with the Declaration of Helsinki.

### 2.2 Molecular studies

P1 and P2 received clinical WES prior to enrollment in the Mount Sinai study to further investigate mt‐aaRSs disorders. WES for P1 was completed at Ambry Genetics (Viejo, CA). Genomic DNA from the proband, mother, and father was isolated from peripheral blood samples, and WES was completed on the family trio. DNA samples were prepared using a SeqCap EZ VCRome 2.0 (Roche NimbleGen, Madison, WI, USA) capture, and 100 base pair (bp), paired end sequencing was performed on an Illumina, San Diego, CA, USA HiSeq 2000 instrument. Mean coverage for the proband was 105.4 with 93.83% of targeted bases with ≥20× coverage. Mean coverage for the mother's sample was 102.31 with 93.56% of targeted bases with ≥20× coverage; mean coverage for the father's sample was 95.78 with 93.6% of targeted bases with ≥20× coverage. WES for P2 was completed at Baylor College of Medicine Medical Genetics Laboratories (Houston, TX). Genomic DNA was prepared from a peripheral blood sample. The exome capture was performed using VCRome 2.1 (Roche NimbleGen, Madison, WI, USA), and 100 bp, paired‐end sequencing was performed on the Illumina HiSeq 2000 platform. The quality control thresholds include mean coverage of targeted bases >100× and >95% of targeted bases covered at >20×. The WES also included mitochondrial genome screening. The mitochondrial genome was amplified by long‐range PCR and subjected to paired‐end library construction. The mean depth of coverage for mitochondrial sequencing was >25,000×.

WES on genomic DNA from affected individual P3 was performed at the Institute of Human Genetics (Munich, Germany) using the SureSelect Human All Exon 50 Mb kit (Agilent, Santa Clara, CA, USA) for in‐solution enrichment followed by sequencing as 75 bp paired‐end runs on a HiSeq2500 (Illumina) as described previously [Haack et al., 2012; Kremer et al., 2016]. This achieved an average of 143‐fold coverage with 97.7% of the exome covered at least 20‐fold.

P4 was investigated by a targeted NGS approach using a customized gene panel (TruSeq Custom Amplicon; Illumina, San Diego, CA, USA) containing genes associated with mitochondrial disorders, according to the procedure recently reported [Legati et al., 2016].

P5 and P7 underwent WES analysis as previously described [Pronicka et al., 2016; Taylor et al., 2014].

P8 and P10 had clinical WES as previously described [Yang et al., 2014]. Analysis of variants followed the guidelines of the American College of Medical Genetics and Genomics [Richards et al., 2015].

Genomic DNA of patients P11‐13 was extracted using standard protocols. P12 underwent clinical WES as previously described [Bastaki et al., 2016].

All the novel sequence variants identified in *VARS2* have been submitted to a public database (https://www.lovd.nl/VARS2).

### 2.3 Histopathological studies

Quadriceps muscle biopsies were obtained from subjects P2, P3, P4, P5, and P6. Transversely orientated, frozen muscle sections (10 μm) were subjected to standard histological and histochemical procedures including sequential cytochrome *c* oxidase (COX)/succinate dehydrogenase (SDH) histochemistry. Electron microscopy of P2 muscle was performed according to standard procedures.

### 2.4 Biochemical studies

MRC complex activities were measured using standard spectrophotometric methods [17] in muscle homogenate of P2, P3, P4, P5, and P6, and digitonin‐treated skin fibroblasts of P4, P5, and P7; complex activities were then normalized to citrate synthase activity, an index of mitochondrial content in the analyzed specimens [Bugiani et al., 2004; Kirby, Thorburn, Turnbull, & Taylor, 2007].

### 2.5 De novo metabolic labeling of mitochondrial encoded proteins

Mitochondrial protein synthesis in P5 cultured cells was performed as previously described [Bruni, Gramegna, Oliveira, Lightowlers, & Chrzanowska‐Lightowlers, 2013]. Briefly, fibroblasts were washed twice in methionine/cysteine‐free DMEM (Sigma, St. Louis, MO, USA). After addition of 0.1 mg/ml emetine, cells were pulsed with [^35^S] methionine/cysteine (PerkinElmer, Waltham, MA, USA) for 1 hr. Aliquots (50 μg) of total cell protein were separated by 15% (w/v) SDS‐PAGE and signals were detected using the Typhoon FLA 9500 PhosphorImager and ImageQuant software (GE Healthcare Life Sciences, Pittsburgh, PA, USA).

### 2.6 Immunoblotting

Lymphoblast samples from P1 and two control lines (C1 and C2) were processed for immunoblotting as previously described [Webb et al., 2015]. The membrane was probed with mouse anti‐OXPHOS cocktail at 1:1,000 (MitoSciences, ab110411, Eugene, OR, USA) and with rabbit anti‐GAPDH at 1:5,000 (Sigma, G9545, St. Louis, MO, USA) used as a loading control. Antibodies were visualized using IRDye 800CW or IRDye 680RD secondary antibodies and the Odyssey Infrared Imaging System (LI‐COR Biosciences, Lincoln, NE, USA).

Total protein was extracted from fibroblasts and muscle tissue of P5 and fibroblasts of P4, separated on 12% SDS‐PAGE gels, transferred to PVDF membranes and analyzed by immunoblotting using primary antibodies to VARS2 (either custom‐made or from MitoSciences, Eugene, OR, USA), NDUFB8, MTCOI, MTCOII, COXIV, SDHA, UQCRC2, ATP5A, and VDAC (all from Abcam, Cambridge, UK). β‐actin (Sigma, G9545, St. Louis, MO, USA), GAPDH (Millipore, Burlington, MA, USA), and α‐tubulin (Abcam) were used as loading controls. Chemiluminescent signals were detected using the Amersham ECL Prime Kit (GE Healthcare Life Sciences, Pittsburgh, PA, USA) and the ChemiDocTM MP Imaging System (Bio‐Rad, Hercules, CA, USA).

### 2.7 Homology modeling

Homology modeling of the mitochondrial human Valine‐tRNA ligase (VARS2, NCBI: NP_001161206.1) in the amino acid interval 130–1,086 was made employing as the template the Protein Data Bank (PDB) structure 1IVS (representing the Valine‐tRNA ligase from *Thermus thermophilus*, which shares 37% amino acid identity with human VARS2). All side chain atoms in the template structure were deleted. Then, the residues of this backbone‐only structure were renamed and renumbered (in PDB format) to the corresponding amino acids in the human VARS2 according to the pairwise sequence alignment shown in the Supp. File S1. All side chains were re‐built and amino acid insertions added using MODELLER (v. 9v17) [Sali et al., 1993] without allowing changes in the model backbone conformation relative to the template structure. The cognate tRNA^Val^ molecule and the Val‐AMP analogue N‐[L‐valyl]‐N’‐adenosyldiaminosulfone (Val‐AMS) co‐crystallized with the template protein were added to the VARS2 model maintaining their binding poses. Finally, the full amino acids of insertions and flanking residues, and the side chains of the remainder of the protein, were energy minimized with the Dreiding force field.

Sequence logos were calculated on the VARS2 multiple sequence alignment as displayed in the MiSynPat database (misynpat.org) including all organisms. Sequence logos were generated using WebLogo [Crooks, Hon, Chandonia, & Brenner, 2004].

## 3 RESULTS

### 3.1 Case reports

**Figure 1:**
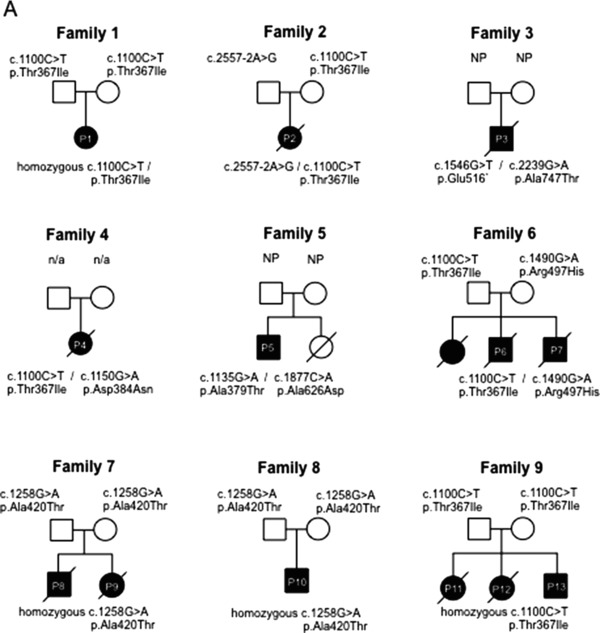
Pedigrees. Pedigrees of the nine families are shown documenting the segregation of alleles

A short clinical description of the patients with biallelic *VARS2* variants is reported below; pedigrees are shown in Figure 1, whereas the main clinical and laboratory findings are reported in Table 1.

**Table 1:** Clinical, laboratory, and biochemical data

						Onset			
Pts	Family	Sex	Ethnicity	Current age/death	Delivery	Age	Neurological signs	MRI	
P1	Family 1	Female	Caucasian (Polish)	Alive at 5 years	Born at full term by normal spontaneous vaginal delivery	From birth	Hypotonia, poor coordination, developmental delay, seizures	Aged 3 years, 6 months: mild, ill‐defined T2/FLAIR hyperintensity in the periventricular white matter bilaterally, cerebral atrophy; small lactate peak at MR	
P2	Family 2	Female	Caucasian	Death at 3.5 months	Born at full term by Cesarean section for breech presentation.	From birth	Hypotonia, hyporeflexia, exaggerated startle, staring episodes, vocal cord paralysis	Aged 2.8 months: moderate to severe diffuse cerebral and cerebellar atrophy. Slightly more focal gliosis identified around the Sylvian fissures bilaterally and within the cerebellum; scattered areas of cortical restricted diffusion as well as subtle thalamic restricted diffusion bilaterally; large lactate peak at MR	
P3	Family 3	Male	Jewish Community	Death at 19 months	Spontaneous delivery at 36 weeks	From birth	Hypotonia, severe stridor, poor sucking; hypertonia of the lower limbs	N/A	
P4	Family 4	Female	Italian	Death at 5 months	IUGR. Uneventful	From birth	Hypotonia, feeding difficulty and psychomotor delay	Cerebellar atrophy, corpus callosum hypotrophy	
P5	Family 5	Male	British	Alive at 18 years	Normal vaginal delivery, at full term	First few months	Developmental delay, ptosis and ophtalmoparesis, generalized epilepsy, fatigue, proximal weakness, dyspraxia	Symmetrical bilateral basal ganglia calcification. Symmetrical increased T2 signal in the peri‐trigonal white matter	
P6	Family 6	MM, male	Polish	Death at 3 months	Born at term by spontaneous delivery	From birth	Hypotonia, stridor and respiratory failure, limbs spasticity	N/A	
P7	Family 6	MK, male	Polish	Death at 9 years	Born at term	From birth	Hypotonia, stridor and respiratory failure, limbs spasticity, epilepsy	Hypoplasia of vermis, mild cerebral atrophy, small symmetric hyperintense changes in thalamus and septum pellucidum	
P8	Family 7	Male	Mexican	Death at 9 days	Born at term	From birth	Hypotonia, feeding difficulty	N/A	
P9	Family 7	Female	Mexican	Death at 3 months	Born at term	From birth	Hypotonia, feeding difficulty	N/A	
P10	Family 8	Male	Mexican	Alive at 3 months	Born at term	From birth	Hypotonia, feeding difficulty, respiratory distress, developmental delay, epilepsy	N/A	
P11	Family 9	Female	Afghanistan	Death at 7 years	Born at term, normal vaginal delivery	From first months	Severe hypotonia, feeding difficulty, psychomotor retardation, nystagmus, intractable epilepsy	1y 1 mo and 2y 4 mo: progressive cerebellar atrophy (cerebellar hemispheres + vermis), signal intensity in dentate nuclei, signal intensity and mild atrophy in thalami, corpus callosum slightly thin	
P12	Family 9	Female	Afghanistan	Death at 8 years	Born at term, normal vaginal delivery	From first months	Hypotonia, feeding difficulty (gastrostomy), psychomotor retardation, limb spasticity, intractable epilepsy	13 months of age: cerebellar atrophy, signal intensity in dentate nuclei and thalami; corpus callosum slightly thin. NO lactate peak in 1H‐MRS	
P13	Family 9	Male	Afghanistan	Alive at 5months	Born at term, normal vaginal delivery	Birth	Hypotonia	Newborn: unilateral mild cerebellar hemispheric hypoplasia	

	Additional clinical signs					Lab tests		OXPHOS	
Pts	Heart	Liver	Kidney	Other	Cause of death	Lactate	Other	Fibroblasts	Muscle
P1	No cardiomyopathy	N/A	Reflux		Alive	N/A	N/A	N/A	N/A
P2	Hypertrophic cardiomyopathy, small patent foramen ovale.	No issues	No issues	Microcephaly (HC at 2%), small nose with flat nasal root, a small mouth with down‐turned corners, micrognathia, and inverted nipples	Not known	Intermittent plasma (from 1.7 to 8.9 mmol/l; nv < 2.1 mmol/l) and urinary elevation	Increased plasma alanine (613 nmol/l)	N/A	Normal
P3	Hypertrophic cardiomyopathy and pulmonary hypertension	No issues	No issues	Microcephaly, craniosynostosis	Not known	Intermittent plasma (3.5–4 mmol/l) and urinary elevation	No issues	N/A	Low CIV activity
P4	Hypertrophic cardiomyopathy	No issues	No issues	microcephaly	Cardiac arrest	Plasma (4.2 mmol/l) and CSF (3 mmol/l; nv < 2.3 mmol/l) elevation	Increased plasma (238 μmol/l; nv 55–145) and CSF (160 μmol/l; nv 45–135) pyruvate	CIV reduction	Normal activities
P5	mild concentric ventricular hypertrophy	N/A	N/A		Alive	N/A	N/A	Normal activity	Combined CI+CIV deficiencies
P6	Hypertrophic cardiomyopathy	No issues	No issues		Not known	Plasma (4.4–8.7 mmol/l) elevation	N/A	NA	Low CIV activity
P7	Hypertrophic cardiomyopathy	No issues	No issues	Cryptorchidism and chronic pancreatitis	Central apnea	Plasma (2.9–10.6 mmol/l) elevation	N/A	Normal activity	NA
P8	Hypertrophic cardiomyopathy	Hepatosplenomegaly	N/A		Severe metabolic acidosis	Plasma elevation	N/A	NA	NA
P9	Hypertrophic cardiomyopathy	Hepatomegaly	N/A		Not known	Plasma elevation	N/A	NA	NA
P10	Hypertrophic cardiomyopathy	N/A	One anuric kidney		Not known	Plasma elevation	N/A	NA	NA
P11	Not evaluated	No issues	No issues	Microcephaly (−3 SD)	Pneumonia	Plasma lactate normal (2.3 mmol/l)	No issues	Not evaluated	Not evaluated
P12	Not evaluated	No issues	No issues	Progressive microcephaly	Pneumonia	Plasma lactate elevation (2.8 mmol/l)	Slightly low carnitine in blood (total 18 umol/l, free 15 umol/l)	Not evaluated	Not evaluated
P13	No cardiomyopathy	None at newborn	None at newborn	Progressive microcephaly	Alive	At newborn: Plasma lactate elevation (4.4 mmol/l), CSF lactate elevation (3.08 mmol/l)	At newborn: Slightly low carnitine in blood (total 15 umol/l, free 13 umol/l)	Not evaluated	Not evaluated

N/A indicates “not available information.”

**P1** is a 5‐year‐old female of Polish ancestry born at 38 weeks gestation by normal vaginal delivery (family 1; Figure 1). Family history is notable for consanguinity; parents are second cousins. She presented with hypotonia and poor suck from birth, followed by developmental delay, poor coordination, dystonic movements and ataxia. At 3.5 years, she was unable to sit, crawl, or walk and her language was limited to fewer than 30 words. At 3 years and 8 months, she presented with seizures, which included generalized tonic‐clonic, focal, and myoclonic types. MRI completed at 11.4 months of age was unrevealing; there were no acute intracranial findings or abnormalities of the orbits or brainstem identified. Evaluation of the parenchyma was limited due to incomplete myelination at this age; the myelination pattern was read as within normal limits for age. The second MRI, performed at 3 years and 6 months of age, revealed T2/FLAIR hyperintensity in the periventricular white matter and cerebellar hemispheres bilaterally, and diffuse cerebral volume loss. MR spectroscopy showed a small doublet peak at 1.3 ppm in voxels over the left parietal white matter and frontal horns, suggesting lactate. An additional MRI, performed at 4 years 9 months revealed more advanced ill‐defined prominent T2/FLAIR hyperintensity in the periventricular white matter as well as multifocal areas of abnormal T2/FLAIR hyperintensity in the cortex and subcortical white matter.

**P2** was a Caucasian female born at 39 weeks gestation by Cesarean section due to breech presentation (family 2; Figure 1). Apgar scores were 7 and 9 at 1 and 5 min, respectively. Shortly after birth, the newborn was transferred to the neonatal intensive care unit for evaluation of stridor. Nasal endoscopy revealed bilateral vocal cord paresis, and the patient was diagnosed with central and obstructive sleep apnea. A tracheostomy was performed for airway protection at 2 months of age. Other symptoms/signs were hypotonia, hyporeflexia, exaggerated startle, staring episodes, and congenital hip dislocation. Echocardiogram completed at 2 months of age revealed moderate to severe biventricular hypertrophy with normal ventricular function and moderate pericardial effusion. Dysmorphic features noted on examination included microcephaly (occipitofrontal circumference [OFC] on 2^nd^ centile), a round, full face, prominent eyes with shallow orbits, a small nose with flat nasal root, a small mouth with down‐turned corners, micrognathia, and inverted nipples. Initial brain MRI completed at 12 days of age was unremarkable, but a second brain MRI performed at 3 months revealed moderate to severe diffuse cerebral and cerebellar atrophy, scattered areas of cortical restricted diffusion, most pronounced around the Sylvian fissures bilaterally, as well as subtle thalamic restricted diffusion bilaterally. MR spectra showed large lactate peaks in the deep gray matter and in the subcortical white matter of the left cerebral hemisphere. The patient died at 3.5 months of age.

**P3** was the son of a non‐consanguineous Jewish couple, born at 36 weeks by spontaneous delivery (family 3; Figure 1). Apgar scores were 7 and 8 at 1 and 5 min, respectively. He presented at birth with severe stridor, difficulty in sucking, dysmorphic features, hypertonia of the lower limbs. Echocardiography revealed hypertrophic cardiomyopathy and severe pulmonary hypertension. Laryngoscopy and bronchoscopy revealed laryngomalacia. The cranium was asymmetric with an OFC of 32 cm. Over the 1st year of life, progressive microcephaly developed due to craniosynostosis (since the age of 5 months the OFC remained at 40 cm). At age 11 months he underwent surgical correction of craniosynostosis. The stridor, cardiomyopathy and pulmonary hypertension improved over the first year of life. However, he continued to feature hypertonia and developmental delay. Brain MRI was not performed. At 19 months, he died suddenly at home during sleep.

**P4** was the second child of healthy, unrelated parents of Italian origin. Family history was unremarkable (family 4; Figure 1). She was born at term following a pregnancy complicated by IUGR in the third trimester; at birth, weight, length, and OFC were on the 3rd centile, Apgar scores were 8 and 9 at 1 and 5 min, respectively. From the first months of life, hypotonia, feeding difficulty and psychomotor delay were reported. She first came to clinical attention at 3 months of age when she suffered from epileptic seizures. Neurological examination showed reduced voluntary movements, no postural control and hypertonia. EEG revealed diffuse abnormalities with poorly organized cerebral rhythms and epileptiform seizure activity. Clobazam and clonazepam treatment was started without obvious benefit. Hypertrophic cardiomyopathy was detected. Cranial MRI revealed hypoplasia of the cerebellum and corpus callosum. A mitochondrial disease was suspected and the patient was referred to our Institute at 4 months of age. Clinical examination showed poor somatic growth and microcephaly (OFC < 3rd centile) and a severe neurological impairment, characterized by absence of head control, response to sounds and language, poor eye contact, hyperreflexia, and hypertonia. Visual evoked potential showed central conduction abnormalities, fundus oculi was normal. The child died at 5 months, because of cardiac arrest.

**Figure 2:**
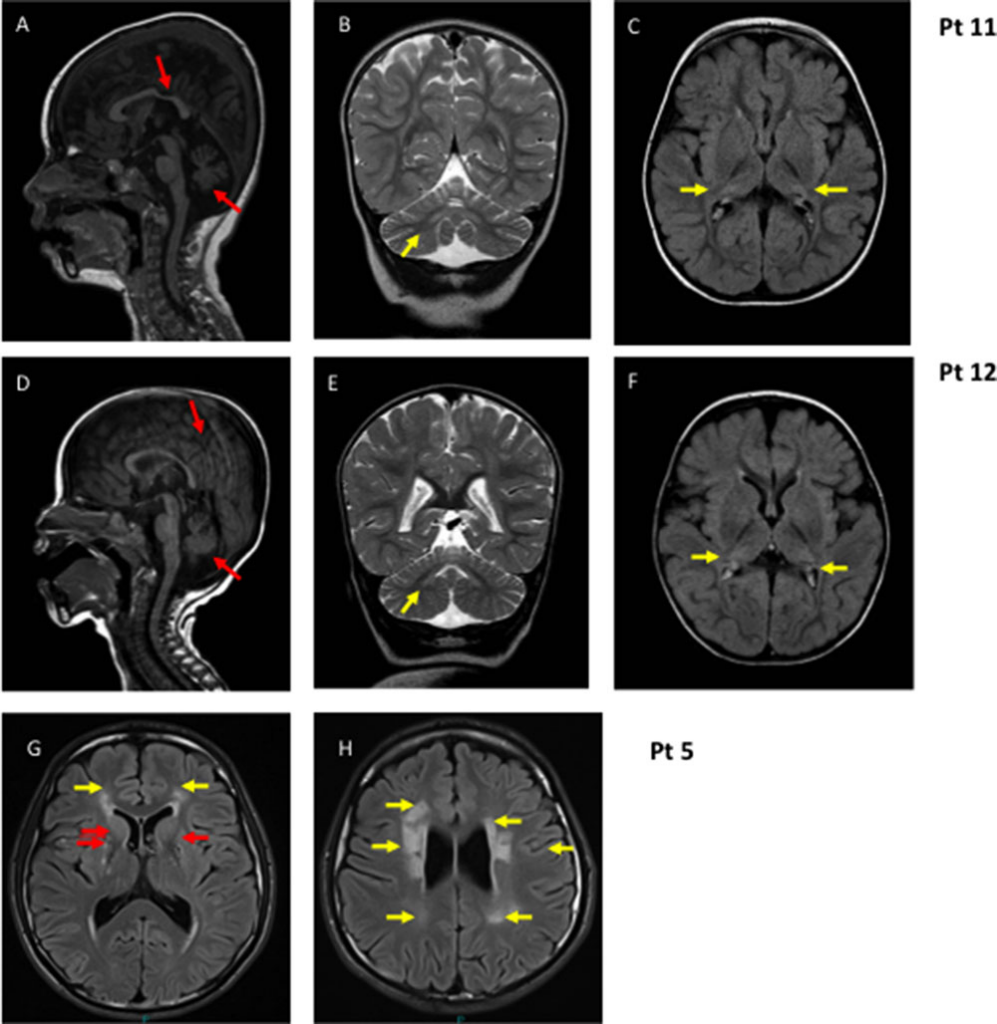
MRI: Sagittal T1 (**A and D**), coronal T2 (**B and E**), and axial flair (**C and F**) images of P11, age 2 years and 4 months, and P12, age 13 months, showing cerebellar atrophy, thinning of the posterior corpus callosum (red/dark arrows), signal intensity in dentate nuclei and pathological signal in the lateral parts of thalami that look smaller (yellow/light arrows). **G and H**: Axial flair images of P5, age 15 years: yellow arrows indicate diffuse symmetrical periventricular white matter abnormality; red arrows indicate basal ganglia calcification

**P5** was born by normal vaginal delivery, at full term (family 5; Figure 1). He was a floppy infant and his clinical history was characterized by psychomotor delay, ptosis and ophthalmoparesis, dysarthria, and dyspraxia. He subsequently developed generalized epileptic seizures that responded favorably to levetiracetam and a slowly progressive fatigue and exercise intolerance. The last neurological examination at 18 years showed restricted range of eye movements in all directions with some sparing of downgaze; bilateral ptosis; generalized hypotonia as well as facial, proximal limb and axial weakness. Cranial MRI and CT demonstrated symmetrical bilateral basal ganglia calcification. In addition, the cranial MRI also revealed a symmetrical increased T2 signal in the peri‐trigonal white matter (Figure 2). Recent echocardiography has revealed mild concentric ventricular hypertrophy. An older sister died at the age of 21 months following pneumonia. She was also floppy and had restricted eye movements with bilateral ptosis. A diagnosis of congenital myasthenia had been considered but she died before this could be definitively investigated.

**P6** was born at term by spontaneous delivery from Polish unrelated parents with a positive genetic history (family 6; Figure 1). Their older daughter presented at birth with hypotonia, laryngeal stridor, respiratory failure, he rapidly developed hypertrophic cardiomyopathy, impaired contractility and died at age of 32 following a cardiac arrest; massive cardiac hypertrophy and fatty liver accumulation were found at autopsy (family 6; Figure 1). The birth weight of P6 was 3,420 g and Apgar score was 10 at 1 min. Fetal and postnatal ECHO was normal. From birth the boy presented with stridor, hypotonia, and respiratory failure, which required mechanical ventilation for 3 weeks. Stridor was still present after extubation but no anatomic abnormality was revealed by two laryngoscopic examinations. During the follow‐up, general clinical condition remained poor and characterized by generalized hypotonia, lack of swallowing reflex, tachycardia, and gallop cardiac rhythm. At 23 days of age, cardiac ultrasound revealed hypertrophy of left ventricle and interventricular septum. Neurological examination revealed poor reactivity with lack of response to visual and auditory stimuli, axial hypotonia, and limb spasticity. The heart size increased rapidly in three consecutive chest radiographs at 20, 50, and 78 days of life. The boy died at 3 months of age.

**P7**, the younger brother of P6 (family 6; Figure 1), was born at term with a body weight of 3,170 g, and Apgar score was 10 at minute 1. Soon after birth he presented with stridor and developed respiratory failure, which required artificial ventilation. Increasing myocardial hypertrophy was observed in three consecutive cardiac ultrasounds. Neurological examination showed poor reactivity, poor facial expression and squint, axial hypotonia with limb spasticity. Hearing examination was normal and ophthalmic fundus unchanged. Head ultrasound did not show any abnormalities. His global clinical condition gradually improved and at 34 days of age the cardiorespiratory picture was stable; the boy was discharged after 2 months of hospitalization. At 7 months of follow‐up, the boy showed psychomotor retardation and episodes of increased muscle tone. Intermittent recurrence of stridor with respiratory difficulty continued. EMG was normal. Brain MRI performed at the age of 8 months showed hypoplasia of the vermis, mild cerebral atrophy and small symmetric T2/FLAIR hyperintense changes in thalamus and septum pellucidum. Since pathological discharges were observed on video‐EEG, symptomatic epilepsy was diagnosed and antiepileptic drugs were administered with good response. At the age of 10 months, he was fully gastrostomy fed. At 4 years the boy had developed chronic respiratory failure and a tracheostomy was performed. Cryptorchidism and chronic pancreatitis were also reported. The patient died at the age of 9 years during an episode of central apnea.

**P8** was a male born to healthy parents of Mexican ancestry (family 7; Figure 1). Pregnancy was uncomplicated and resulted in spontaneous delivery at term. The patient presented with hypotonia and poor sucking at birth. Respiratory failure followed soon after birth and ultrasound revealed hypertrophic cardiomyopathy with biventricular dilation and thickening of the ventricular walls, as well as hepatosplenomegaly. The child suffered a severe metabolic acidosis including high plasma lactate and died on the ninth day of life. **P9** was a female full‐sibling to P8, born after her brother's death, whose clinical course was remarkably similar and died at 3 months of age (family 7; Figure 1).

**P10** was a male born following an uncomplicated pregnancy to healthy parents of Mexican descent (family 8; Figure 1). The child experienced hypotonia of all limbs and feeding difficulties from birth. Respiratory distress also occurred in the first few days of life and the child was found to have hypertrophic cardiomyopathy. Seizures began in the first month of life and continued despite treatment with antiepileptic drugs. The patient also experienced severe metabolic acidosis with persistently increased lactate and was noted to have a single anuric kidney.

**P11** was the first child of a consanguineous healthy couple of Afghan origin (family 9; Figure 1). She was born at term (birth weight 3,300 g, length 51 cm), and had no neonatal problems. She was referred for investigations at the age of 6 months because of poor head control. On examination, she was noted to be hypotonic, microcephalic, and developmentally delayed. Nystagmus was noticed by the age of 1 year and at the age of 1 year 3 months a brain MRI showed cerebellar atrophy, slight thinning of the posterior corpus callosum and pathological FLAIR hyperintense signal in the thalami. At the age of 2 years, when her epileptic seizures started, she was able to say a few words and turn over, but never learned to crawl. She developed intractable multifocal epilepsy with prolonged seizures. A repeat MRI at the age of 2 years 4 months showed progression of the cerebellar atrophy, involving both cerebellar hemispheres and vermis, and FLAIR/T2 hyperintense signal in dentate nuclei and thalami (Figure 2). Her disease progressed with regression of skills and feeding difficulties evident by the age of 4 years. She died of pneumonia at the age of 7 years.

**P12**, sister of patient 11, the second child of the family, was a term baby (birth weight 3,450 g/length 49 cm/OFC 35 cm, Apgar score 9 at 1 min) (family 9; Figure 1). At the age of 6 months, when first investigated for delayed motor development, she was hypotonic with poor head control and microcephaly (−2 SD). Brain MRI at the age of 1 year 1 month showed cerebellar atrophy and slightly thinning of the posterior corpus callosum (Figure 2). A 1H‐MRS from the thalamus showed no lactate peak and EEG showed no epileptiform discharges. Epileptic seizures started with status epilepticus at the age of 2 years 1 month and EEG revealed multifocal spikes, spike‐slow waves and general disturbance. At that age she was alert, able to say few words, able to roll over and catch toys. By the age of 4 years her disease had progressed, skills had regressed, she had developed limb spasticity and needed a gastrostomy for feeding. Epilepsy was intractable. She died of pneumonia at the age of 8 years.

**P13**, brother of patients 11 and 12, the third child of the family, was diagnosed with *VARS2* mutations antenatally (family 9; Figure 1). He was born at term (birth weight 3,810 g, length 52.5 cm/OFC 34.0 cm (−1 SD), Apgar score 9 at 1 min). Neonatal brain MRI showed unilateral cerebellar hypoplasia. No neonatal problems were noticed. At 5 months of age, his head growth was slowed to −3 SD and he was hypotonic without any signs of cardiomyopathy in heart ultrasound.

### 3.2 Morphological studies

Histochemistry of P3 and P4 muscle samples was normal, whereas mild, unspecific myopathic changes in P5 and signs of neurogenic atrophy in P6 were reported (not shown). In P5, COX‐deficient/SDH‐positive fibers were evident (not shown).

Electron microscopy of P2 muscle disclosed relatively normal myofibrillar architecture, myofiber nuclei, sarcolemmal membranes, and basement membranes; there was abnormal/excessive accumulation of glycogen (non‐membrane‐bound) both intermyofibrillary and especially subsarcolemmal, which has a variably granular appearance to very focally filamentous appearance. The mitochondria appeared relatively decreased in number (sparse overall) and size, with mostly unremarkable morphology although some are distorted or atypical (with atypical cristae) (not shown).

### 3.3 Autopsy findings

For P2, post‐mortem examination of the heart revealed severe biventricular hypertrophy and mildly asymmetrical hypertrophy of the ventricular septum; microscopic sections showed prominent and diffuse myocyte vacuolar degeneration. Neuropathological evaluation revealed a subacute and acute necrotizing encephalopathy consistent with Leigh syndrome with lesions evident in the thalamus and cortex, having typical histopathology of vacuolated neuropil with gliosis and prominent vasculature. Cerebellar cortical degeneration was also noted; the substantia nigra appeared relatively spared (not shown).

In P6, post‐mortem examination showed hydrothorax and hydropericardium, and left ventricle hypertrophy with immature cardiac cells.

### 3.4 Biochemical studies

**Figure 3:**
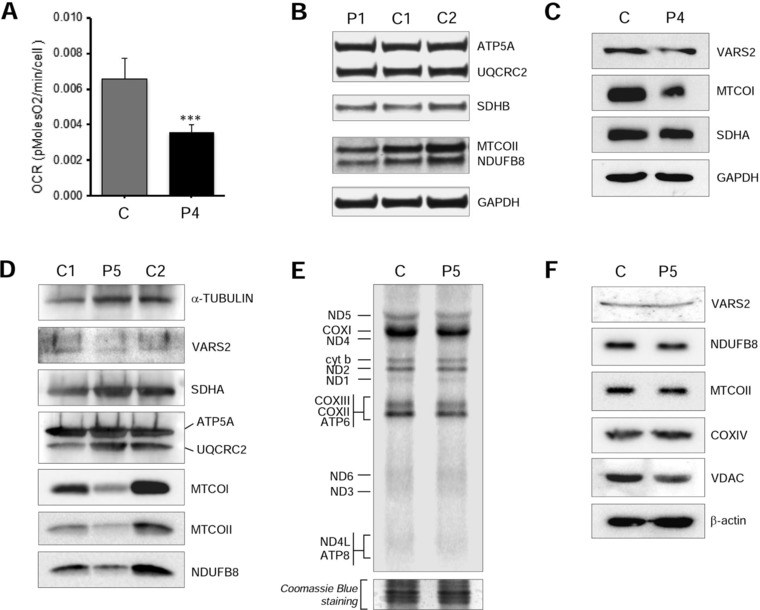
Functional studies. **A**: Micro‐oxygraphy performed in P4 and control fibroblasts cultured in galactose medium. *Y*‐axis values correspond to the maximal respiration rate, expressed as pMolesO_2_/min/cell. Data are represented as mean ± SD. Two‐tail, paired *t*‐test was applied for statistical significance (***P < 0.001). **B**: Western blot analysis of P1 lymphoblasts using anti‐OXPHOS cocktail (ATP5A, UQCRC2, SDHB, MTCOII, NDUFB8) and anti‐GAPDH antibodies. **C**: Western blot analysis of P4 fibroblasts using antibodies against VARS2, mitochondrial encoded Complex IV subunit 1 (MTCOI), Complex II subunit A (SDHA), and GAPDH, the latter being used as a loading control. **D**: Western blot analysis of P5 muscle sample using antibodies against VARS2, Complex I subunit (NDUFB8), Complex II subunit (SDHA), Complex III core protein II (UQCRC2), Complex IV subunits (MTCOI, MTCOII), Complex V subunit (ATP5A) and alpha‐tubulin, the latter being used as a loading control. **E**: De novo metabolic labeling in P5 and control fibroblasts. Separated on 15% PAA gel. Coomassie blue stain as loading control. **F**: Western blot analysis of P5 fibroblasts using antibodies against VARS2, NDUFB8, Complex IV subunits (MTCOII, COXIV), VDAC and beta‐actin, the last two being used as loading controls

The activities of all the MRC complexes were within control range in P5 and P7 fibroblasts, whereas a partial reduction of CIV/CS (69% of mean control value) was observed in P4 fibroblasts. In these cells respiratory capacity, assessed by Seahorse micro‐scale oxygraphy, was normal in standard, glucose medium but was decreased in galactose‐medium, a condition that forces cells to use OXPHOS to produce ATP rather than glycolysis (Figure 3A).

MRC complex activities in muscle samples disclosed a combined CI and CIV reduction in P5 (60% of mean control values), a CIV reduction in P3 (17% of mean control) and P6 (25% of mean control), whereas were normal in P2 and P4 muscles.

### 3.5 WES and Sanger validation

Details of the identified *VARS2* variants are provided in Table 2. All variants are numbered according to NM_001167734 reference sequence.

**Table 2:** Information about the identified *VARS2* mutations

	cDNA (NM_001167734.1)	Protein (NP_001161206.1)	Domain	Allele inheritance	ExAC frequency (%)	Polyphen2, HumVar score	SIFT, score	ACMG
P1	Homozygous c.1100C > T	p.Thr367Ile	synt/editing	P + M	0.0026	Probably D.0.998	Deleterious0.00	Pathogenic
P2	c.2557‐2A > G	Aberrant splicing	anticodon	P	0.0228	/	/	Pathogenic
	c.1100C > T	p.Thr367Ile	synt/editing	M	0.0026	Probably D.0.998	Deleterious0.00	Pathogenic
P3	c.1546G > T	p.Glu516*	aatRNA synt	?	0.0117	/	/	Pathogenic
	c.2239G > A	p.Ala747Thr	aatRNA synt		0.0009	Probably D.0.995	Deleterious 0.01	Likely Pathogenic
P4	c.1100C > T	p.Thr367Ile	synt/editing	^	0.0026	Probably D.0.998	Deleterious 0.00	Pathogenic
	c.1150G > A	p.Asp384Asn	synt/editing		0.0009	Probably D.1.000	Deleterious 0.00	Pathogenic
P5	c.1135G > A	p.Ala379Thr	synt/editing	?	n.r.	Probably D.0.999	Deleterious 0.00	Pathogenic
	c.1877C > A	p.Ala626Asp	aatRNA synt		0.0009	Possibly D.0.517	Deleterious 0.04	Pathogenic
P6	c.1100C > T	p.Thr367Ile	synt/editing	P	0.0026	Probably D.0.998	Deleterious 0.00	Pathogenic
	c.1490G > A	p.Arg497His	aatRNA synt	M	0.0025	Probably D.1.000	Deleterious 0.01	Pathogenic
P7	c.1100C > T	p.Thr367Ile	synt/editing	P	0.0026	Probably D.0.998	Deleterious 0.00	Pathogenic
	c.1490G > A	p.Arg497His	aatRNA synt	M	0.0025	Probably D.1.000	Deleterious 0.01	Pathogenic
P8	homozygous c.1258G > A	p.Ala420Thr	synt/editing	P + M	0.0312	Probably D.0.996	Deleterious 0.05	Pathogenic
P9	homozygous c.1258G > A	p.Ala420Thr	synt/editing	P + M	0.0312	Probably D.0.996	Deleterious 0.05	Pathogenic
P10	homozygous c.1258G > A	p.Ala420Thr	synt/editing	P + M	0.0312	Probably D.0.996	Deleterious 0.05	Pathogenic
P11	homozygous c.1100C > T	p.Thr367Ile	synt/editing	P + M	0.0026	Probably D.0.998	Deleterious 0.00	Pathogenic
P12	homozygous c.1100C > T	p.Thr367Ile	synt/editing	P + M	0.0026	Probably D 0.998	Deleterious 0.00	Pathogenic
P13	homozygous c.1100C > T	p.Thr367Ile	synt/editing	P + M	0.0026	Probably D.0.998	Deleterious 0.00	Pathogenic

P, paternal; M, maternal; ?, inheritance not ascertained; ^, variants appear on different alleles, parental DNA not available; probably D, probably damaging; n.r., not reported.

Web URLs and parameters of software used for predictions are as follows: Polyphen v.2, https://genetics.bwh.harvard.edu/pph2 . Minimal alignment length 100; Minimal identity in alignment 0.5; Maximal gap length in alignment 20; Threshold for contacts 6 Å. The PolyPhen‐2 score ranges from 0.0 (tolerated) to 1.0 (damaging). SIFT, https://sift.bii.a-star.edu.sg/www/SIFT_seq_submit2.html.

Selected database: Uniprot‐SwissProt 2010_09; Median conservation of sequences: 3.00; Remove sequences more than 90% identical to query. SIFT scores ≤0.05 correspond to amino acid substitutions predicted to affect protein function.

A homozygous, reported pathogenic, c.1100C > T (p.T367I) *VARS2* variant (ClinVar: RCV000129937.4) was detected in P1. Both parents were heterozygous for this variant. In P2 compound heterozygous *VARS2* variants, c.2556‐2A > G and c.1100C > T (p.T367I), were identified. Sanger sequencing confirmed the proband to be heterozygous for both variants, and identified the mother to be heterozygous for the c.1100C > T variant and the father to be heterozygous for the c.2556‐2A > G variant.

In P3 after excluding previously annotated single nucleotide changes occurring with high frequency in the population, prioritizing homozygous or compound heterozygous variants with functional impact (i.e., non‐synonymous variants and changes affecting splice sites) as expected for a recessively inherited trait, WES identified two compound heterozygous variants in *VARS2*: c.1546G > T (p.Glu516*) and 2239G > A (p.Ala747Thr).

After filtering procedure similar to WES (frequency threshold > 1%) in P4 we identified two variants in *VARS2*: c.1100C > T (p.Thr367Ile) and c.1150G > A (p.Asp384Asn). The analysis of the single NGS reads revealed that the two variants are present on different alleles, confirming the compound heterozygous state of the patient. Sanger sequence confirmed the presence of the variants.

In P5 and P7, *VARS2* mutations were identified by WES and confirmed by Sanger sequencing as previously described [Pronicka et al., 2016; Taylor et al., 2014]. P5 was found heterozygous for the c.1135G > A (p.Ala379Thr) and c.1877C > A (p.Ala626Asp) mutations whereas P7 resulted compound heterozygous for c.1100C > T (p.Thr367Ile) and c.1490G > A (p.Arg497His).

In P6, older affected brother of P7, the same p.Thr367Ile and p.Arg497His mutations were identified by Sanger sequencing.

Clinical WES was conducted on P8, which reported the child was homozygous for c.1258G > A (p.Ala420Thr). Sanger sequencing confirmed the presence of the mutation in the child and in the proband's sibling P9, as well as the heterozygous state of the parents. Patient P10 also e clinical WES: although he was reported to be unrelated to family of P8‐P9, the same homozygous c.1258G > A (p.Ala420Thr) variant was identified.

Clinical WES was also conducted on P12, and it detected the same homozygous c.1100C > T (p.Thr367Ile) variant in *VARS2* as in P1. Sanger sequencing confirmed the presence of the mutation in the proband and in her affected siblings (P11 and P13), as well as their parents’ heterozygosity.

### 3.6 Functional studies

In order to assess the effect of the identified *VARS2* variants on protein levels, immunoblotting was performed using available patient samples (P1 lymphoblasts, P4 fibroblasts, and P5 muscle). NDUFB8 and MTCOII protein levels were reduced in P1 lymphoblasts compared with control samples (Figure 3B). A decrease in steady state level of VARS2 protein was observed in P4 fibroblasts compared with control lines (Figure 3C); furthermore, a reduction in MTCOI levels was present in P4, whereas SDHA levels were unaffected (Figure 3C). P5 muscle showed normal VARS2 steady state levels but decreased amounts of Complex I (NDUFB8) and Complex IV (MTCOI and MTCOII) subunits compared with a control muscle (Figure 3D).

Next we determined the consequence of the *VARS2* mutations on mitochondrial translation by performing de novo metabolic labeling in patient and control fibroblasts. Data analysis in P5 fibroblasts compared with aged‐matched control cells indicated that mutant VARS2 caused no significant changes in the rate of mitochondrial translation (Figure 3E). Immunoblotting analysis was performed on the same fibroblasts and also showed no differences in VARS2 levels or steady‐state levels of Complexes I and IV subunit (Figure 3F), consistent with the normal mitochondrial protein synthesis and reports of other mutated mitochondrial aa‐tRNA synthetases, where the consequential biochemical defect is usually expressed in muscle but not in cultured fibroblasts [Almalki et al., 2014].

## 4 DISCUSSION

Mitochondrial diseases due to defects in mt‐aaRS are emerging as an important category of mitochondrial disorders. Despite reporting of a strict genotype–phenotype association for many mt‐aaRS, in some cases mutations in the same gene have been associated with very different clinical phenotypes; for example, pathogenic variants in *AARS2* (encoding for alanyl‐tRNA synthetase) are associated either with infantile mitochondrial cardiomyopathy [Götz et al., 2011] or leukodystrophy with ovarian failure [Dallabona et al., 2014]. Other examples are the different clinical phenotypes caused by *FARS2* [Almalki et al., 2014; Elo et al., 2012], *LARS2* mutations [Pierce et al., 2013; Riley et al., 2016], or *MARS2* mutations [Bayat et al., 2012; Webb et al., 2015]. It remains poorly understood why genetic defects in mt‐aaRSs that are ubiquitous enzymes involved in mitochondrial translation, can cause such different phenotypes in which specific tissues or organs are affected. Different hypotheses have been postulated to explain this variability, taking into account the tissue‐related amino acid concentrations, the different cell susceptibility to impaired mitochondrial protein synthesis, variable protein turnover in different tissues or the multiple and still unknown functions of mt‐aaRSs. For this reason, describing the phenotype related to a specific mt‐aaRSs becomes both important as well as intriguing.

Recently, Baertling et al. (2017) reported a patient harboring compound heterozygous pathogenic *VARS2* variants presenting with neonatal encephalocardiomyopathy. Here we describe the clinical and biochemical features of 13 additional *VARS2* patients from 9 unrelated families (Table 1, Supp. Tables S1, and S2). Almost all patients presented with a phenotype characterized by severe early‐onset encephalopathy with hypotonia, stridor and respiratory failure. Hypertrophic cardiomyopathy is a common feature of this disorder, although it was absent in P1 who presented with the severe, early‐onset clinical picture described in the other patients. Cardiomyopathy was not investigated in P11 or P12. P11 and P12 presented a severe encephalopathy with refractory epilepsy, dying at 7 and 8 years, respectively, whereas P13, whose molecular diagnosis was detected antenatally, had elevated lactate and minor changes at brain MRI already at birth, showed slowed head growth and hypotonia, but was otherwise symptom‐free at 5 months of age. P1 and P13 harbored the same homozygous *VARS2* variant, and do not present cardiomyopathy; the first patient reported by Diodato et al. (2014a,b), homozygous for the same variant, did not present cardiomyopathy either. These data could suggest that the c.1100C > T (p.Thr367Ile) variant could have a lesser effect to the heart. Epilepsy seems to be a common feature of *VARS2*‐related mitochondrial disease, configuring clinical pictures of variable severity (Supp. Table S2). Seizures appeared later in life in the other patient described with a long survival (P7). P5 presented a less severe course characterized by developmental delay, epilepsy, ptosis, ophthalmoparesis and myopathy. In P5 a mild concentric ventricular hypertrophy was discovered in his late teens. In our cohort there was no significant or specific involvement of other organs, apart from kidney reflux reported in P1, anuric kidney in P10, cryptorchidism and recurrent pancreatitis described in P7, and hepatosplenomegaly in P8 and P9. Interestingly, recurrent pancreatitis has been recently described in a patient with another defect of mitochondrial protein synthesis, namely recessive mutations in *RMND1*, which codes for a protein involved in mitochondrial translation (Required for Meiotic Nuclear Division 1, *S. cerevisiae* homolog, MIM# 614917) [Ng et al., 2016]. Nevertheless, we cannot exclude a possible role of antiepileptic drugs in determining the recurrent pancreatitis. Lactic acidosis, where assessed, is a common feature. Notably, pulmonary hypertension, described in P3, was described in patients harboring *TMEM70* (MIM# 612418) pathogenic variants [Catteruccia et al., 2014]. MRI patterns were rather non‐specific, showing variable cerebral and cerebellar atrophy, white matter abnormalities and basal ganglia involvement; corpus callosum hypotrophy, frequently seen in mitochondrial disorders, was described in the patients reported by Diodato et al. (2014ba,b) and recently by Baertling et al. (2017).

The biochemical phenotype is mainly represented by a combined defect in muscle, as in P3, P5, and P6, and almost always normal in fibroblasts. Interestingly, P4 showed a partial Complex IV defect in fibroblasts, whereas biochemical analyses were normal in muscle. WB analyses performed in P4 fibroblasts and P5 muscle confirmed the biochemical results. Additionally, decreased protein levels for NDUFB8, representing Complex I, and MTCOII, representing Complex IV, were noted in P1 lymphoblasts. Overall, our cohort strengthens and develops the previous biochemical description of *VARS2* patients [Diodato et al., 2014ba,b; Taylor et al., 2014], and the biochemical phenotype caused by mt‐aaRS mutations [Diodato et al., 2014ba,b], which is frequently normal in fibroblasts cell lines.

The mutant *VARS2* allele c.1100C > T (NM 001167734.1) is recurrent in our population, either in homozygosity (P1; P11‐P13) or in compound heterozygosity (P2, P4, P5, P6‐7). The c.1100C > T allele was present in homozygosity in the first patient of Italian ancestry [Diodato et al., 2014ba,b], and was also present in a Greek patient [Baertling et al., 2017]. In the present cohort, P1, P6, P7 patients are Polish, P11‐P13 are Afghan, whereas P4 is Italian. The absence of a shared ethnic origin suggests either different independent events or a very ancient common founder. The *VARS2* variant c.1258G > A was observed in three patients from two independent families of Mexican origin. All three patients were homozygous for this allele, despite no reports of consanguinity in the families. The allele has a frequency of 0.2 % in the ExAC Latino population, which is the highest allele frequency among the mutations reported here. This allele is reported in 0.01% of ExAC Europeans but is not reported in African or Asian ExAC populations and as such may represent an allele that has expanded in the Latino population.

**Figure 4:**
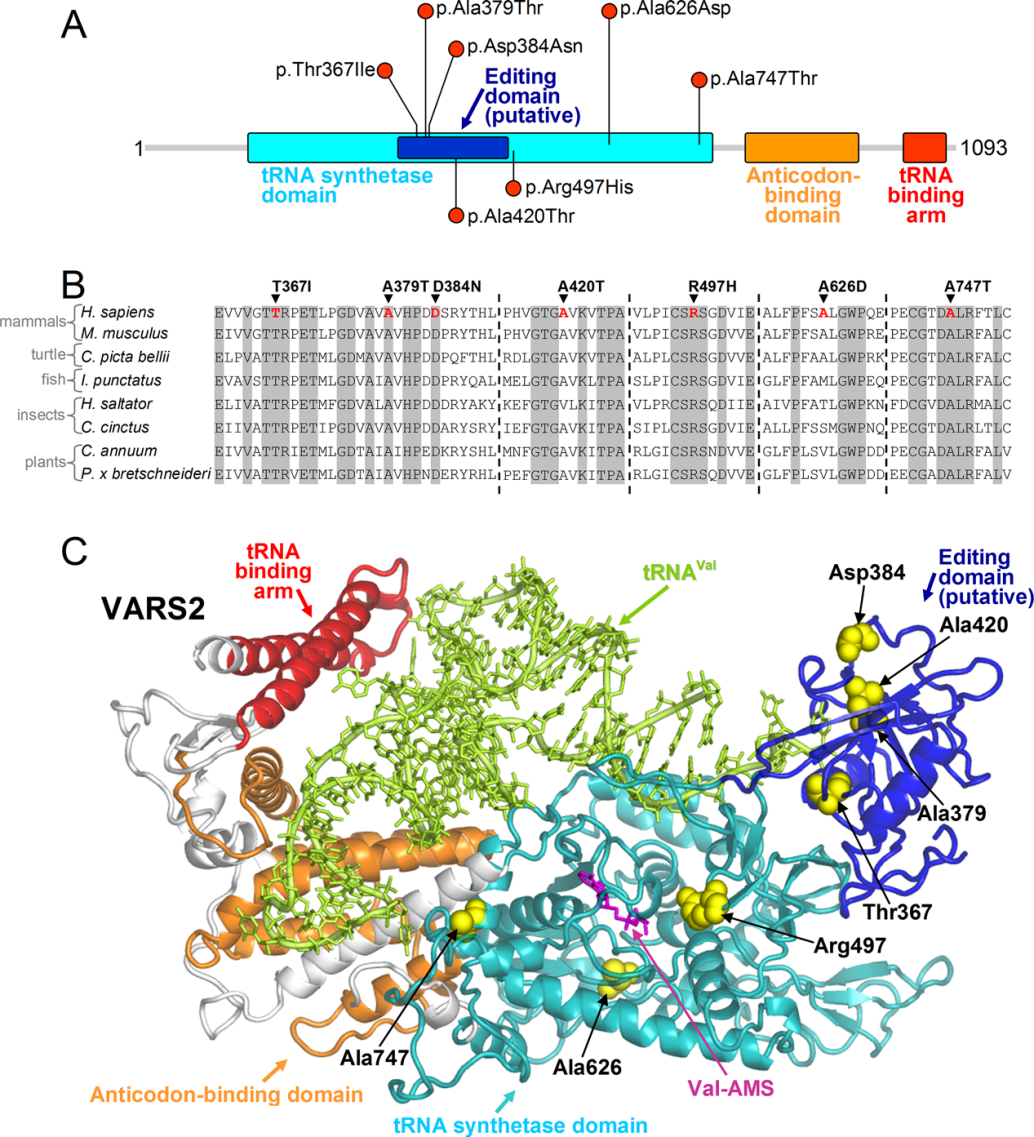
Scheme and molecular model of VARS2 protein. **A**: schematic view of VARS2 protein. All the mutations reported here fall in the tRNA synthetase domain. **B**: VARS2 sequence alignment among representative eukaryotes (*H. sapiens*, NM_001167734.1; *M. musculus*, NM_175137.4; *C. picta bellii*, XM_008176875.1; *I. punctatus*, XM_017481440.1; *H. saltator*, XM_011150470.1; *C. cinctus*, XM_015730429.1; *C. annuum*, XM_016687220.1; *P. x bretschneideri*, XM_009342992.2) around the sites of the missense mutations discussed in the text (Thr367Ile, Ala379Thr, Asp384Asn, Ala420Thr, Arg497His, Ala626Asp, Ala747Thr). Residues that are invariant in this group of eukaryotes are shown in gray. **C**: homology model of VARS2. VARS2 protein (ribbons in different colors for the various functional regions), the residues affected by the missense mutations (yellow spheres), the bound cognate tRNA (tRNA‐Val, light green ribbons and sticks), and the Val‐AMP analogue (Val‐AMS, magenta sticks) are shown. The pathogenic mechanism of these mutations can be inferred from their location: Thr367Ile, Ala379Thr, Asp384Asn, Ala420Thr, and Ala747Thr occur at protein sites relevant for the binding of the tRNA molecule, whereas the Arg497His and Ala626Asp mutations affect the binding pocket of the cognate valine

Five out of the six missense mutations affect residues located in regions important for the interaction of VARS2 with the cognate tRNA (p.Thr367Ile, p.Ala379Thr, p.Asp384Asn, p.Ala420Thr, and p.Ala747Thr) (Figure 4). Crucially, most of these mutations involve residues that are highly conserved among phylogenetically distant organisms (Ala420 presents valine or cysteine as alternative residues implying that this site requires conserved hydrophobicity) (Figure 4B and Suppl. Figure S2). This underscores the functional importance of these sites, allowing us to suggest, as a common pathogenic consequence of the above non‐conserved amino acid replacements, a defective binding of tRNA.

The p.Arg497His and p.Ala626Asp mutations fall near the binding pocket of the cognate valine. The first causes the replacement of an invariant arginine with a histidine that can also acquire a positive charge through protonation but presenting much lower p*K*_a_ than the protonated arginine and thus the latter has stronger capability to maintain a positive charge also inside the protein environment. In addition, the rigid aromatic ring of the histidine side chain does not reproduce the interactions of the flexible side chain of the native arginine. Although the site of the Ala626Asp mutation is apparently not highly conserved, the alternative residues found in other organisms are either hydrophobic or serine or threonine: in contrast with aspartic acid, all these amino acids contain aliphatic portions that can preserve the local multiple interactions as engaged by Ala626 (Suppl. Figure S3). Furthermore, in the alignment (Figure 4B and Suppl. Figure S2), this site never hosts a negatively charged residue like that introduced with the Ala626Asp replacement. This can be understood considering that such variant would cause salt‐bridge shuffling, owing to its proximity to the ionic pair Arg274‐Asp635. Based on these observations, Arg497His and Ala626Asp mutations are both expected to modify the conformation of the valine binding region and thus to impair enzyme function.

## 5 CONCLUSIONS

Here we describe the clinical and biochemical phenotype of 13 patients harboring bi‐allelic pathogenic *VARS2* variants. The common phenotype is characterized by a severe, early onset cardioencephalomyopathy associated with a combined OXPHOS defect in muscle. The brain MRI did not show any characteristic recognizable pattern across patients. Patient P1 has no evidence of cardiomyopathy; she and P5 (mild, later onset cardiomyopathy) are the only subjects who remain alive at ages 5 and 18 years, respectively. It is reasonable to infer from our cohort's data that a poor prognosis seems to correlate with the severity of cardiomyopathy/myopathy. P11 and P12, in which cardiomyopathy was not investigated, presented a severe encephalopathy with refractory epilepsy and died at 7 and 8 years, respectively. Indeed patient P5 also had a relatively mild neurological phenotype. These last observations are in line with the extreme clinical variability of mt‐aaRS associated phenotypes, even though the described VARS2‐associated phenotype looks rather homogenous. Structural and functional analyses clearly support the pathogenic role of the identified variants. The series of patients described here, reinforces previous findings and further delineates the range of clinical observations caused by mutations in *VARS2*, which should be investigated in early‐onset mitochondrial encephalomyopathies or encephalocardiomyopathies.

## Supporting information

Supp. File S1, Pairwise sequence alignment: Pairwise sequence alignment (clustal format) employed for the homology modelling of the mitochondrial human Valine‐tRNA ligase (VARS2, NCBI: NP_001161206.1) in the residue interval 130‐1086 and the Valine‐tRNA ligase from Thermus thermophilus (PDB 1IVS).
Suppl. Fig. S2, Sequence logos: Amino acid frequency calculated on the VARS2 multiple sequence alignment as available in the MiSynPat database, including all 105 organisms ranging from mammals to bacteria, around the sites of the missense mutations presented in this study (Thr367Ile, Ala379Thr, Asp384Asn, Ala420Thr, Arg497His, Ala626Asp, Ala747Thr).
Supp. Fig. S3, Enlarged view of VARS2 model around Ala626: The interactions of Ala626 side chain with the hydrophobic moieties of Val210, Cys276 and Phe622, which are disrupted by the p.Ala626Asp pathogenic variant, are shown. The introduction of the negatively charged Asp residue should also cause salt‐bridge shuffling with the nearby Arg274‐Asp635 ionic pair. The p.Ala626Asp variant is thus expected to cause conformational changes in proximity of the binding pocket of the cognate valine suggesting impaired enzymatic transfer of this ligand to the tRNA.
Supplementary Table S1: Frequencies of the main clinical and MRI features in our *VARS2* mutant patients
Supplementary Table S2: Echocardiography and epilepsy feature**s**
