# Supplementary material for: Clinical, biochemical, and genetic features associated with VARS2‐related mitochondrial disease
Source: Hum Mutat. 2018 Feb 7;39(4):563–78. doi: 10.1002/humu.23398 (PMC5873438; doi:10.1002/humu.23398)
Supplement: Supplementary file 1 — Supp. File S1, Pairwise sequence alignment: Pairwise sequence alignment (clustal format) employed for the homology modelling of the mitochondrial human Valine‐tRNA ligase (VARS2, NCBI: NP_001161206.1) in the residue interval 130‐1086 and the Valine‐tRNA ligase from Thermus thermophilus (PDB 1IVS). Suppl. Fig. S2, Sequence logos: Amino acid frequency calculated on the VARS2 multiple sequence alignment as available in the MiSynPat database, including all 105 organisms ranging from mammals to bacteria, around the sites of the missense mutations presented in this study (Thr367Ile, Ala379Thr, Asp384Asn, Ala420Thr, Arg497His, Ala626Asp, Ala747Thr). Supp. Fig. S3, Enlarged view of VARS2 model around Ala626: The interactions of Ala626 side chain with the hydrophobic moieties of Val210, Cys276 and Phe622, which are disrupted by the p.Ala626Asp pathogenic variant, are shown. The introduction of the negatively charged Asp residue should also cause salt‐bridge shuffling with the nearby Arg274‐Asp635 ionic pair. The p.Ala626Asp variant is thus expected to cause conformational changes in proximity of the binding pocket of the cognate valine suggesting impaired enzymatic transfer of this ligand to the tRNA. Supplementary Table S1: Frequencies of the main clinical and MRI features in our VARS2 mutant patients Supplementary Table S2: Echocardiography and epilepsy features [file HUMU-39-563-s001.doc]

**Supp. File S1**, **Pairwise sequence alignment:** Pairwise sequence alignment (clustal format) employed for the homology modelling of the mitochondrial human Valine-tRNA ligase (VARS2, NCBI: NP_001161206.1) in the residue interval 130-1086 and the Valine-tRNA ligase from Thermus thermophilus (PDB 1IVS).

---------------------------------------------------------------------------------------------------------

Color scheme highlighting functional regions:

■ tRNA synthetases, residues 140-765 (including the putative editing domain)

■ Editing domain, putative, residues 342-490

■ Anticodon-binding domain, residues 809-961

■ Valyl tRNA synthetase tRNA binding arm, residues 1021-1079

---------------------------------------------------------------------------------------------------------

VARS2 (human) LPPAYSPRYVEAAWYPWWVREGFFKPEYQARLPQATGETFSMCIPPPNVTGSLHIGHALT

ValRS (1IVS-A) LPKAYDPKSVEPKWAEKWAKNPFVA------NPKSGKPPFVIFMPPPNVTGSLHMGHALD

** **.*: **. * *.:: *. *:: .* : :**********:****

VARS2 (human) VAIQDALVRWHRMRGDQVLWVPGSDHAGIATQAVVEKQLWKERGVRRHELSREAFLREVW

ValRS (1IVS-A) NSLQDALIRYKRMRGFEAVWLPGTDHAGIATQVVVERLLLKE-GKTRHDLGREKFLERVW

::****:*::**** :.:*:**:********.***: * ** * **:*.** **..**

VARS2 (human) QWKEAKGGEICEQLRALGASLDWDRECFTMDVGSSVAVTEAFVRLYKAGLLYRNHQLVNW

ValRS (1IVS-A) QWKEESGGTILKQLKRLGASADWSREAFTMDEKRSRAVRYAFSRYYHEGLAYRAPRLVNW

**** .** * :**: **** **.**.**** * ** ** * *: ** ** :****

VARS2 (human) SCALRSAISDIEVENRPLPGHTQLRLPGCPTPVSFGLLFSVAFPVDGEPDAEVVVGTTRP

ValRS (1IVS-A) CPRCETTLSDLEVETE----------------PTPGKLYTLRYEVEGGGFIEIATV--RP

. .:::**:***.. : * *::: : *:* *:.. **

VARS2 (human) ETLPGDVAVAVHPDDSRYTHLHGRQLRHPLMGQPLPLITDYAVQPHVGTGAVKVTPAHSP

ValRS (1IVS-A) ETVFADQAIAVHPEDERYRHLLGKRARIPLTEVWIPILADPAVEKDFGTGALKVTPAHDP

**: .* *:****:*.** ** *:: * ** :*:::* **: ..****:******.*

VARS2 (human) ADAEMGARHGLSPLNVIAEDGTMTSLCG-DWLQGLHRFVAREKIMSVLSEWGLFRGLQNH

ValRS (1IVS-A) LDYEIGERHGLKPVSVINLEGRMEGERVPEALRGLDRFEARRKAVELFREAGHLVKEEDY

* *:* ****.*:.** :* * . : *:**.** **.* :.:: * * : :::

VARS2 (human) PMVLPICSRSGDVIEYLLKNQWFVRCQEMGARAAKAVESGALELSPSFHQKNWQHWFSHI

ValRS (1IVS-A) TIALATCSRCGTPIEYAIFPQWWLRMRPLAEEVLKGLRRGDIAFVPERWKKVNMDWLENV

.:.*. ***.* *** : **::* : :. .. *.:. * : : *. :* .*:.::

VARS2 (human) GDWCVSRQLWWGHQIPAYLVVEDHAQGEEDCWVVGRSEAEAREVAAELTGRPGAELTLER

ValRS (1IVS-A) KDWNISRQLWWGHQIPAWYCED------CQAVNVPRPERYLED---PTSCEACGSPRLKR

** :************: : :. * *.* .: : .. .. *:*

VARS2 (human) DPDVLDTWFSSALFPFSALGWPQETPDLARFYPLSLLETGSDLLLFWVGRMVMLGTQLTG

ValRS (1IVS-A) DEDVFDTWFSSALWPLSTLGWPEETEDLKAFYPGDVLVTGYDILFLWVSRMEVSGYHFMG

* **:********:*:*:****:** ** *** .:* ** *:*::**.** : * :: *

VARS2 (human) QLPFSKVLLHPMVRDRQGRKMSKSLGNVLDPRDIISGVEMQVLQEKLRSGNLDPAELAIV

ValRS (1IVS-A) ERPFKTVLLHGLVLDEKGQKMSKSKGNVIDPLEMVERY----------------------

: **..**** :* *.:*:***** ***:** :::.

VARS2 (human) AAAQKKDFPHGIPECGTDALRFTLCSHGVQAGDLHLSVSEVQSCRHFCNKIWNALRFILN

ValRS (1IVS-A) ---------------GADALRFALIYLATGGQDIRLDLRWLEMARNFANKLYNAARFVLL

*:*****:* .. . *::*.: :: .*:*.**::** **:*

VARS2 (human) ALGEKFVPQPAEELSPSSPMDAWILSRLALAAQECERGFLTRELSLVTHALHHFWLHNLC

ValRS (1IVS-A) SREGFQ-----AKEDTPTLADRFMRSRLSRGVEEITALYEALDLAQAAREVYELVWSEFC

: : ...: * :: ***: ..:* : : :*: .:: ::.: ::*

VARS2 (human) DVYLEAVKPVLWHSPRPLGPPQVLFSCADLGLRLLAPLMPFLAEELWQRLPPRPGCPPAP

ValRS (1IVS-A) DWYLEAAKPALKAG--NAHTLRTLEEVLAVLLKLLHPMMPFLTSELYQALT------GKE

* ****.**.* . . :.* . : *:** *:****:.**:* *.

VARS2 (human) SISVAPYPSACSLEHWRQPELERRFSRVQEVVQVLRALRATYQLTKARPRVLLQSSEPGD

ValRS (1IVS-A) ELALEAWP----EPGGRDEEAERAFEALKQAVTAVRALKAEAGLP-PAQEVRVYLEG--E

.::: .:* *: * ** *. :::.* .:***:* *. . .* : . :

VARS2 (human) QGLFEAFLEPLGTLGYCGAVGLLPPGAAAPSGWAQAPLSDTAQVYMELQGLVDPQIQLPL

ValRS (1IVS-A) TAPVEENLEVFRFLSR---ADLLPE----RPAKALVKAMPRVTARMPLEGLLDVEEWRRR

. .* ** : *. ..*** .. * . . . * *:**:* :

VARS2 (human) LAARRYKLQKQLDSLTARTPSEGEAGTQRQQKLSSLQLELSKLDKAASHLRQLMDEPP

ValRS (1IVS-A) QEKRLKELLALAERSQRKLASPGFREKAPKEVVEAEEARLKENLEQAERIREALSQIG

* :* : : .* * . :: :.: : .*.: : *.::*: :.:

**Suppl. Fig. S2, Sequence logos**: Amino acid frequency calculated on the VARS2 multiple sequence alignment as available in the MiSynPat database, including all 105 organisms ranging from mammals to bacteria, around the sites of the missense mutations presented in this study (Thr367Ile, Ala379Thr, Asp384Asn, Ala420Thr, Arg497His, Ala626Asp, Ala747Thr).

**
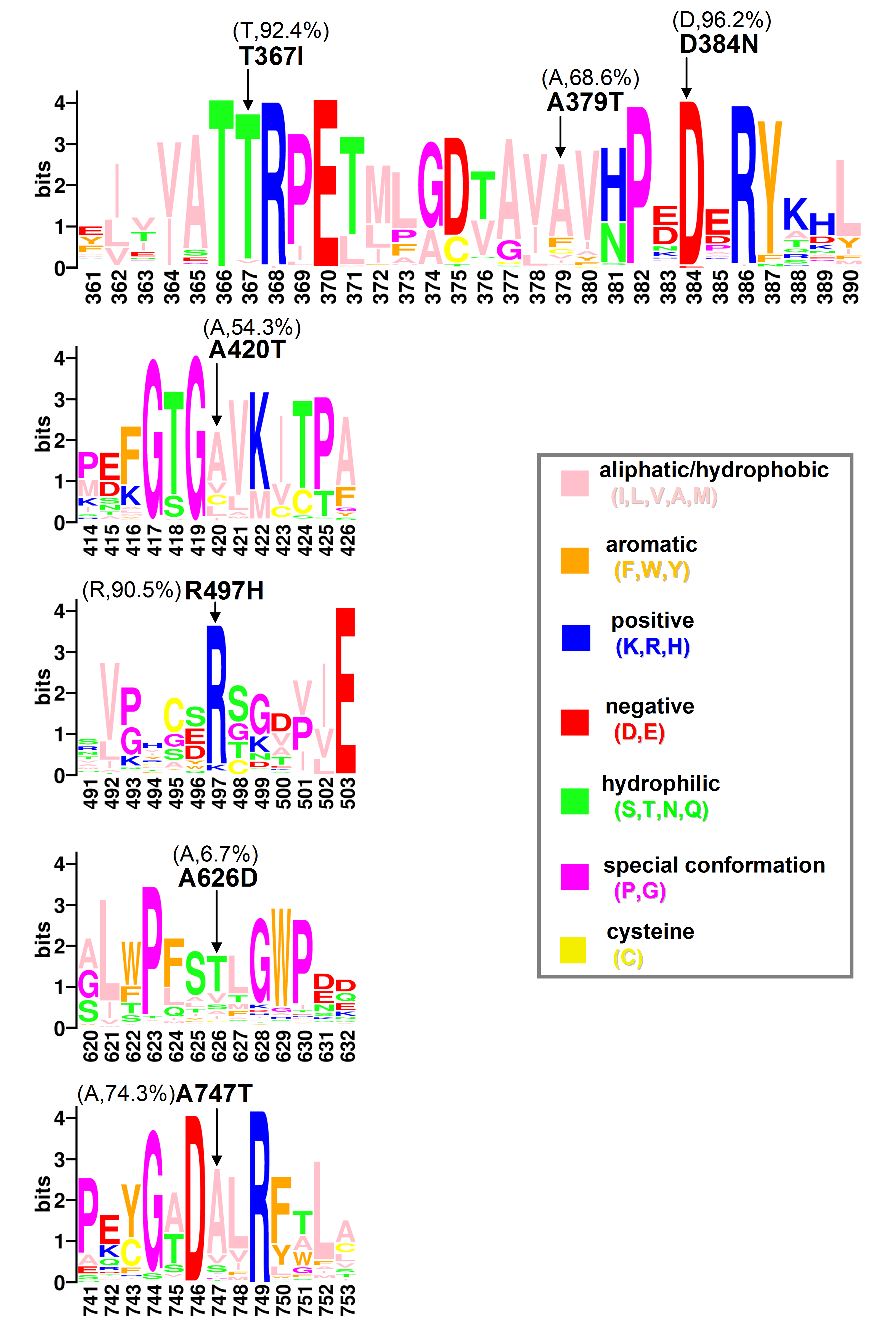
**

**Supp. Fig. S3**, **Enlarged view of VARS2 model around Ala626**: The interactions of Ala626 side chain with the hydrophobic moieties of Val210, Cys276 and Phe622, which are disrupted by the p.Ala626Asp pathogenic variant, are shown. The introduction of the negatively charged Asp residue should also cause salt-bridge shuffling with the nearby Arg274-Asp635 ionic pair. The p.Ala626Asp variant is thus expected to cause conformational changes in proximity of the binding pocket of the cognate valine suggesting impaired enzymatic transfer of this ligand to the tRNA.


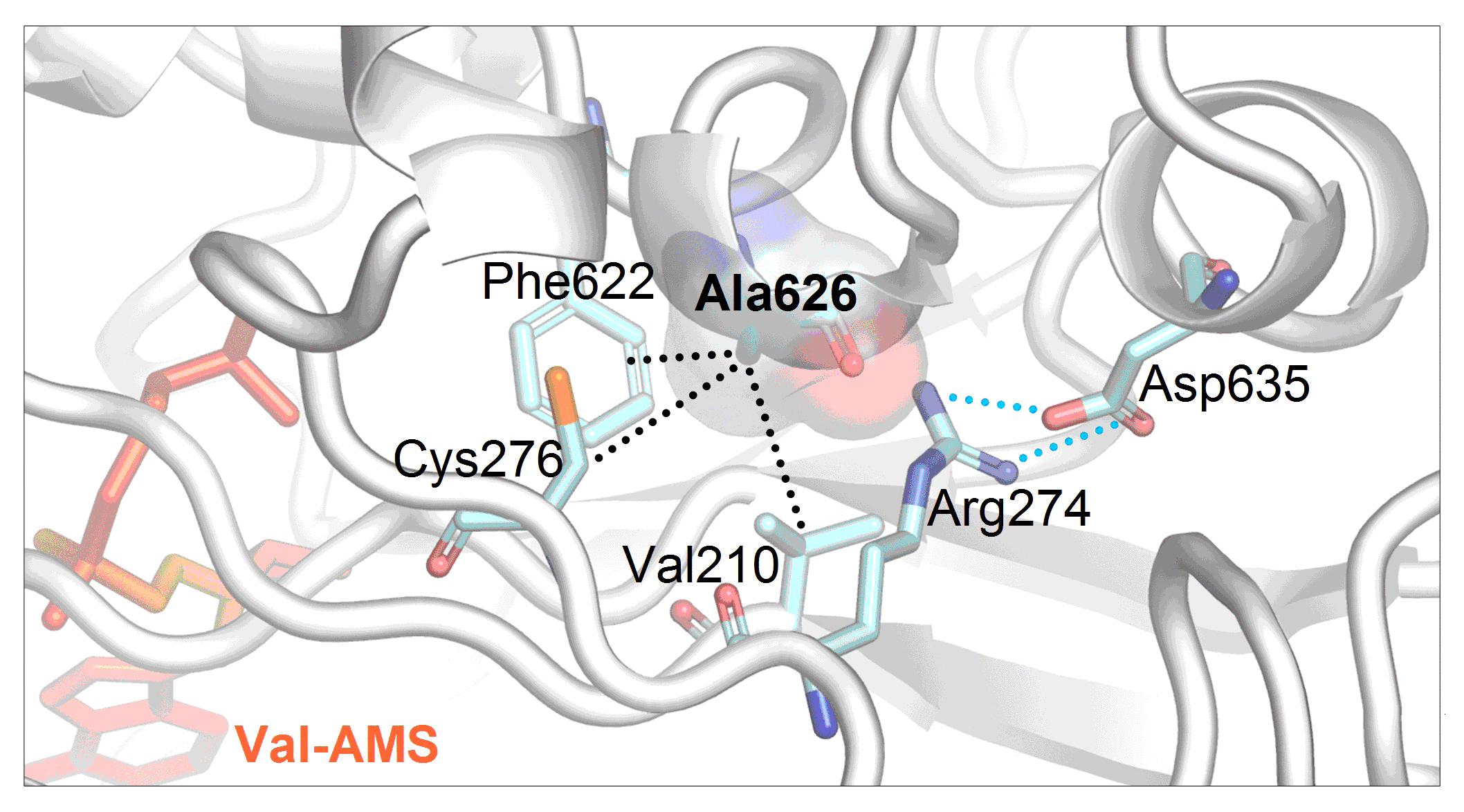


**Supplementary Table S1: Frequencies of the main clinical and MRI features in our *VARS2* mutant patients**

|  | **Clinical signs** | | | | | | | **MRI** | | | | |
| --- | --- | --- | --- | --- | --- | --- | --- | --- | --- | --- | --- | --- |
|  | **Hypotonia** | **Hypertrophic cardiomyopathy** | **Psychomotor delay** | **Feeding difficulty** | **Epilepsy** | **Stridor** | **Lactic acidosis** | **Cerebellar atrophy** | **Thalami involvement** | **Basal ganglia calcifications** | **White matter changes** | **Cerebral atrophy** |
| **Pts** | 12/13 | 9/11 | 6/13 | 6/13 | 6/13 | 4/13 | 11/13 | 6/8 | 4/8 | 1/8 | 3/8 | 3/8 |

**Supplementary Table S2: Echocardiography and epilepsy features**

| **Pts** | **Ecocardiography features (including LV thickness)** | **Epilepsy type** |
| --- | --- | --- |
| **P1** | No cardiomyopathy | tonic-clonic, focal and myoclonic seizures |
| **P2** | N/A | No epilepsy |
| **P3** | Performed at 1.5 years:  - Borderline increased thickness of LV (7 mm)  - Tricuspid valve regurgitation: mild to moderate, grade 1-2/4  -Very mild pulmonary hypertension | No epilepsy |
| **P4** | N/A | Multifocal and diffuse EEG abnormalities |
| **P5** | Performed at the age of 15 years:  -LV septum measured 10.9mm and posterior wall 11mm (top end of normal range for body surface area)  -LV internal dimension was 48mm (diastole) and 31mm (systole) | Generalized epileptic seizures |
| **P6** | N/A | No epilepsy |
| **P7** | N/A | No information available |
| **P8** | N/A | No epilepsy |
| **P9** | N/A | No epilepsy |
| **P10** | N/A | No information available |
| **P11** | N/A | Multifocal, symptomatic epilepsy, therapy-resistant |
| **P12** | N/A | Multifocal, symptomatic epilepsy, therapy-resistant |
| **P13** | No cardiomyopathy, LV 25(+0.7SD)/15 mm | No |
